# How can we make interventions more ‘acceptable’ in mental health?

**DOI:** 10.1136/bmjment-2025-302337

**Published:** 2026-02-27

**Authors:** Thomas Kabir

**Affiliations:** 1 Department of Experimental Psychology, University of Oxford, Oxford, UK; 2 Department of Psychiatry, University of Oxford, Oxford, UK

**Keywords:** Psychiatry, Psychology, Mental Health

## Abstract

Are we properly assessing the acceptability of mental health interventions from a service user’s point of view? A treatment can be efficacious and effective but still not acceptable to a service user. For example, someone with psychosis may find a treatment that improves symptoms but causes significant weight gain and sedation unacceptable. Despite significant progress being made in developing safe and effective interventions for mental health problems, a notable proportion of people remain in need. The reasons for this are complex. Part of the problem could be an inadequate focus on how ‘acceptable’ interventions really are to service users given their views and specific circumstances.

Treatment acceptability has been a rather poorly defined concept. This has led to researchers using several methods to assess their own ideas about what service user acceptability is. This can include methods such as using recruitment and withdrawal data. But such data may not be true markers of acceptability to a service user.

Current approaches to assessing acceptability need improvement. Existing acceptability questionnaire measures are not widely used and are often developed for specialist settings. Crucially, very few have had any stated involvement of people with mental health conditions in their development. This approach risks not listening to service users’ voices fully.

Developing better ways of understanding and assessing service users’ views of treatment acceptability in mental health could have many benefits—including empowering individual service users to identify interventions that they can and cannot accept. It is time to better understand what ‘acceptable’ really means in mental health.

## Introduction

 We have effective and seemingly robust ways of developing mental health interventions. In recent years, good progress has been made in developing safe and effective interventions for mental health problems. A recent highlight is the approval by the U.S. Food and Drug Administration (FDA) of Cobenfy for use in the USA.^1^

And yet some people are still left in need. For example, only around 50% of people are in symptomatic remission 4 years after experiencing first episode psychosis.^2^ Looking further afield, it has been recognised that despite advances, remission rates for people with eating disorders remain ‘suboptimal’.^3 4^ Engagement and drop-out rates with digital interventions can be problematic.^5^ In short, a case could be made that we do not have enough effective treatments. And that some of the treatments that we do have are not taken up in a sustained way by enough people. The reasons for all this are complex and the subject of much research.

I suggest that part of the problem behind these different issues is that we do not truly look closely enough at how ‘acceptable’ interventions really are from a service user’s point of view. If we were able to empower service users to select interventions that are acceptable to them, then remission rates may ultimately be somewhat improved.

This is not easy. Indeed, do we even really know what ‘acceptability’ means in a context such as mental health where treatment can be given on a compulsory basis? To be more specific, someone may have a treatment preference in case they are detained under the UK’s Mental Health Act. They may wish to take some medications, but not others.

A possible solution to this would be to first ask this service user what would be acceptable to them and then to complete an Advance Choice Document.^6^ The treatment(s) could then potentially be acceptable to a service user even if they are given involuntarily.

### What is ‘treatment acceptability’?

In my opinion, the conceptualisation and measurement of treatment acceptability is a rather underdeveloped area of research. Treatment acceptability measures generally ask a service user to weigh up all aspects of an intervention to get an overall idea of how ‘acceptable’ something is to them. Different definitions of treatment acceptability have been proposed over the years. But none appear to be widely used. Indeed, Sekhon and colleagues noted that ‘defining acceptability is not a straightforward matter. Definitions within the healthcare literature vary considerably, highlighting the ambiguity of the concept’.^7^

The same authors propose a general definition: ‘acceptability is a multifaceted construct that reflects the extent to which people delivering or receiving a healthcare intervention consider it to be appropriate, based on anticipated or experienced cognitive and emotional responses to the intervention’.^7^

Using this definition, Sekhon and colleagues have produced the Theoretical Framework of Acceptability (TFA) questionnaire which is based around seven constructs: affective attitude, burden, ethicality, intervention coherence, opportunity costs, perceived effectiveness and self-efficacy.^8^

### How treatment acceptability has been assessed

A sizeable body of literature does exist that looks at acceptability of interventions. These can take the form of feasibility studies. At first glance, the methods used to assess acceptability appear to vary widely. These include bespoke questionnaires,^9^ using recruitment and withdrawal data,^10^ and qualitative methods.^11^ Satisfaction and other existing measures are used. But these measures tend to only map on to some of the constructs identified by Sekhon and colleagues. In other words, we may only be partially assessing acceptability. Additionally, a specific criterion that is used to determine if something is acceptable or not is sometimes lacking.

Specific treatment acceptability questionnaire measures do exist. A 2020 systematic review found 32 such measures.^12^ Most of these measures were developed for use in specialist settings such as schools. Around five measures have been adapted for general use across any intervention type. The latest is the TFA.

### What are the problems with current approaches?

None of these specific treatment acceptability measures that we have are widely used in research or clinical practice. Few have had any stated involvement of people with mental health conditions in their development. It is encouraging that there has been service user involvement in a range of outcome measures that do cover some of the acceptability constructs identified by Sekhon.^13^ But these measures are not acceptability measures and are not presented as such.

The methods that we do have to assess acceptability may not consider sufficiently service users’ individual preferences and circumstances. For example, a service user with depression who is trying to conceive may find an antidepressant unacceptable.

The variety of methods used to assess acceptability makes it difficult to compare different interventions directly. Qualitative methods are used to assess acceptability. But these methods can be labour intensive and difficult to compare results.

Crucially, while more research into the methods used to assess the acceptability of mental health interventions is needed, it does seem unlikely that researchers are using measures that cover all the constructs that have been identified by Sekhon and others. Recruitment and withdrawal data, for example, will not necessarily indicate if the service user found the approach to be helpful.

### What could be the benefits of assessing acceptability?

I suggest that there could be benefits to assessing acceptability directly from the service user perspectives in both research and routine clinical care.

In general terms, assessing acceptability in this way means that it would be a service user-reported outcome. This is important as some approaches rely on clinician-reported data. Second, acceptability is a composite concept. This offers us a potentially more comprehensive and valid endpoint than relying primarily on changes in symptoms or recruitment data. If we had ways of reliably and consistently assessing acceptability, then it would allow us and service users to compare between different intervention types.

The assessment of acceptability has clear benefits for researchers as it helps researchers to understand if service users are likely to accept the intervention at all. Second, if there are problems (such as the intervention being too burdensome), then revisions can be made, as is already routine practice. An example would be a digital therapy for depression. If daily use was too burdensome for service users (even if doing so would be best as measured by symptom reduction), then less frequent use can be considered.

In terms of service users, the prospective assessment of acceptability potentially gives them the opportunity to choose something that is acceptable to them. Perhaps a telling example comes from a randomized controlled trial (RCT) for generalised anxiety disorder from 2017.^14^ In this pilot study, service users were offered either a medication or psychological therapy. The study was closed due to poor recruitment. The reasons given for this are that ‘Three quarters of those service users identified as possibly suitable for the trial declined to take part; the great majority did so because they did not want to be randomly assigned to receive medication’.^15^ A prospective assessment of service user acceptability may have identified this issue.

There could be benefits in routine care as well. Taking the example of someone initiating a pharmacological treatment. If acceptability was low, then the dose could be changed, or a different medication could be prescribed. Support around side effects should additionally be given. Of course, this is something that is already done intuitively by clinicians. But this approach may give an additional service user-centred way of identifying if help is needed.

Looking at acceptability retrospectively is likely to be helpful to regulators such as the National Institute for Health and Care Excellence (NICE) or the FDA, and commissioners of healthcare services. If service users find certain treatments to be particularly acceptable, then this is useful information to consider when commissioning or approving treatments for use.

### What can be done?

A sensible way forward would be to work with service users to see if the definition proposed by Sekhon and colleagues fully holds for circumstances specific to mental health, such as when people undergo involuntary treatment. The point is to find a robust and acceptable definition. Carrying out a review of the ways that acceptability has been assessed in the literature seems prudent.

A next step would be to co-produce a consistent and feasible way(s) of assessing the acceptability of mental health interventions.

Doing this would help us to directly compare the acceptability of different intervention types. Significant care would need to be taken to ensure that any approach is not burdensome for service users.

A third phase of this work would be how best to implement the assessment of acceptability in both research and routine clinical care.

## Summary

Are we really hearing the voices of service users clearly enough? I have argued that routinely assessing treatment acceptability potentially has a range of benefits to service users, clinicians, researchers, regulators and commissioners of services. We need to better conceptualise what ‘acceptable’ means in mental health. We need to co-produce with service users’ ways of assessing acceptability that consider their individual views and circumstances. In the end, it is the service user who makes the decision about if something is acceptable. We must therefore need to listen to them carefully and ensure that their voice is heard clearly.
